# Malaria parasite detection increases during pregnancy in wild chimpanzees

**DOI:** 10.1186/1475-2875-13-413

**Published:** 2014-10-20

**Authors:** Hélène M De Nys, Sébastien Calvignac-Spencer, Christophe Boesch, Pierre Dorny, Roman M Wittig, Roger Mundry, Fabian H Leendertz

**Affiliations:** Epidemiology of Highly Pathogenic Microorganisms, Robert Koch-Institute, Nordufer 20, 13353 Berlin, Germany; Department of Primatology, Max-Planck-Institute for Evolutionary Anthropology, Deutscher Platz 6, 04103 Leipzig, Germany; Laboratory of Parasitology, Faculty of Veterinary Medicine, Ghent University, Salisburylaan 133, 9820 Merelbeke, Belgium; Department of Biomedical Sciences, Institute of Tropical Medicine, Nationalestraat 155, 2000 Antwerp, Belgium; Taï Chimpanzee Project, Centre Suisse de Recherche Scientifique, BP1301, Abidjan, Côte d’Ivoire; Max-Planck-Institute for Evolutionary Anthropology, Deutscher Platz 6, 04103 Leipzig, Germany

**Keywords:** Malaria, Chimpanzee, *Plasmodium*, Pregnancy, Epidemiology, Great ape

## Abstract

**Background:**

The diversity of malaria parasites (*Plasmodium* sp.) infecting chimpanzees (*Pan troglodytes*) and their close relatedness with those infecting humans is well documented. However, their biology is still largely unexplored and there is a need for baseline epidemiological data. Here, the effect of pregnancy, a well-known risk factor for malaria in humans, on the susceptibility of female chimpanzees to malaria infection was investigated.

**Methods:**

A series of 384 faecal samples collected during 40 pregnancies and 36 post-pregnancies from three habituated groups of wild chimpanzees in the Taï National Park, Côte d’Ivoire, were tested. Samples were tested for malaria parasites by polymerase chain reaction (PCR) and sequencing. Data were analysed using a generalized linear mixed model.

**Results:**

Probability of malaria parasite detection significantly increased towards the end of pregnancy and decreased with the age of the mother.

**Conclusions:**

This study provides evidence that susceptibility to malaria parasite infection increases during pregnancy, and, as shown before, in younger individuals, which points towards similar dynamics of malaria parasite infection in human and chimpanzee populations and raises questions about the effects of such infections on pregnancy outcome and offspring morbidity/mortality.

**Electronic supplementary material:**

The online version of this article (doi:10.1186/1475-2875-13-413) contains supplementary material, which is available to authorized users.

## Background

Chimpanzees (*Pan troglodytes*), gorillas (*Gorilla gorilla*) and humans are hosts to a similar diversity of malaria parasites (*Plasmodium* spp.) [1, 2]. The phylogenetic relationships of African great ape (AGA) *Plasmodium* spp. have been described in detail but many aspects of their biology are poorly understood. For example, while the high prevalence of the infection in wild AGA and the absence of recorded death imputable to these parasites point at a weak pathogenicity [3, 4], the fact that all *Plasmodium* spp. infecting humans cause morbidity and that experimental infection of chimpanzees evidenced malaria-like symptoms [5] raises the possibility of some impact on wild AGA population health.

In fact, even the basic features of the epidemiology of AGA malaria parasites are only beginning to be understood. A naturally appealing strategy is to verify whether well-known epidemiologic characteristics of human malaria are mirrored by AGA malaria parasites. For example, a key feature of human malaria in hyperendemic areas is that infants are faced with both a higher prevalence of parasitaemia and a higher risk of mortality than adults (especially when it comes to malignant malaria) [6]. Using non-invasive samples acquired from a wild chimpanzee community, De Nys *et al.* recently showed that the probability of detection of malaria parasites decreases with age [4]. This suggests that heavily exposed chimpanzees progressively become resistant to infection and/or high parasitaemia, which mirrors observations made on humans.

In humans, another group at risk are pregnant women (e.g., [7]). They are more vulnerable to both malaria infection and clinical malaria than non-pregnant women, particularly during the first pregnancies [8, 9]. The main adverse outcomes resulting from malaria in pregnancy in endemic areas are maternal anemia (possibly resulting in maternal mortality), low birth weight due to intra-uterine growth retardation and/or pre-term birth, and possibly miscarriages [10]. Infants born from women with malaria also seem to face an increased susceptibility to malaria infection during infancy [11]. Malaria in pregnancy thus contributes significantly to pregnant women and child morbidity and mortality in endemic areas [11, 12].

Pregnancy being a key determinant for reproductive success and infant survival, investigating the relationship between pregnancy and malaria in wild chimpanzee populations is an important step to improve the understanding of what this parasitic infection represents for these populations’ fitness. Here, the results of a mixed longitudinal and cross-sectional study during which the probabilities of malaria parasite infection in wild chimpanzees throughout pregnancy and post-pregnancy were determined, are reported.

## Methods

### Sample and gestational data collection

**Figure 1:**
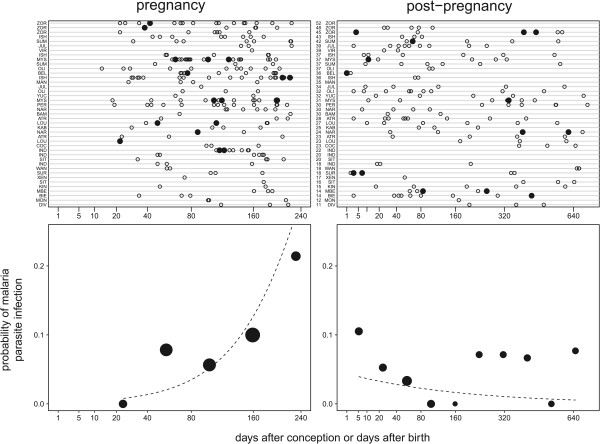
**Probability of malaria parasite infection as a function of time during and after pregnancy.** The upper panel shows data points per pregnancy and/or post-pregnancy over time (filled dots: malaria positive, open dots: malaria negative). Pregnancies and post pregnancies are referred to by the mother’s name (three-letter abbreviation) and ranked by increasing age of the mother at infant birth. The lower panel shows the probability of malaria parasite infection as a function of gestational age (bottom left) or age of the infant after birth (bottom right; age in days). The points depict the proportion of samples tested positive, the area of the points corresponds to the number of samples tested in the respective age class and the dashed lines show the probability of malaria positivity per 200,000 units mitochondrial mammal DNA.

A total of 384 faecal samples collected from wild chimpanzees in the Taï National Park, Côte d’Ivoire, during the course of 40 pregnancies (N = 222) and 36 post-pregnancy periods (N = 162) were tested by polymerase chain reaction (PCR) for malaria parasites (Figure 1). Faecal samples were collected between 2001 and 2013 from 27 different females, aged 11 to 52 years at the time of sampling and belonging to three habituated groups of the Taï Chimpanzee Project (N = 16, 6 and 5, respectively). Group sizes varied from 16 to 54 individuals. Samples were collected straight after defecation, stored in liquid nitrogen at latest 12 hours after collection and then preserved at -80°C. Note that, for the same population, we already reported an overall average faecal detection rate of 35% and a clear decrease in detection probability with age [4].

All pregnancies included in the study resulted in the birth of a viable newborn except for one stillbirth; all post-pregnancy samples were collected from lactating females up to 718 days after birth to avoid miscarriages or stillbirths resulting in unnoticed pregnancies. To estimate gestational ages, conception dates were calculated by subtracting the average chimpanzee pregnancy duration of 225 days [13], which was also estimated for the study population (T Deschner, pers comm), from infant birth dates. However, based on oestrus data, the stillbirth was estimated to be a premature birth at approximately 6.5 months and its conception date was estimated according to information on oestrus. To make sure that samples were obtained from pregnant females, only samples collected after the latest possible conception date were used (based on the shortest estimated pregnancy duration of 208 days [13]). For five births, only the month was known. For these cases the last day of the birth month was used as birth date to calculate conception date, and samples collected during the birth month were excluded.

### Molecular analysis

DNA was extracted from pea-sized faecal samples using the EURxGene MATRIX stool DNA purification kit (Roboklon, Berlin, Germany). Screening for malaria parasites was done using a nested qPCR targeting a 90 bp fragment in the non-coding region of the mitochondrial DNA. Subsequently positive samples were further screened with a semi-nested standard PCR (350 bp) targeting a fragment comprising the 3’ end of the cytochrome oxidase 1 gene, a short intergenic region and the 5’ end of the cytochrome *b* gene [4]. Semi-nested PCR products for which no distinct sequences were obtained were cloned using a Topo TA cloning kit (Invitrogen™, Karlsruhe, Germany). Following colony PCR, positive colony PCR products were purified and sequenced according to the Sanger’s method. Samples were considered positive in either of the following cases: a) *Plasmodium* sequences were obtained directly from semi-nested PCR products (N = 19) or from clones of semi-nested PCR products (N = 1); b) samples positive for the nested qPCR (90 bp) but for which no sequences were obtained (N = 19), i.e. negative for the semi-nested 350 bp PCR or subsequent sequencing and cloning, retested positive by nested standard PCR using nested qPCR primers (see Additional file 1). These positive samples (N = 8) were then tested for monkey species DNA to avoid false positives resulting from contamination of faeces with *Hepatocystis* sp. after prey (monkey) consumption and were only considered positive if negative for monkey DNA (N = 7); c) *Hepatocystis* sp. sequences were obtained (N = 5) and were negative (N = 1) when tested for monkey DNA. To test for monkey DNA, a pan-mammal assay targeting a 130 bp fragment of the mitochondrial 16S gene was used with blocking primers for human and chimpanzee DNA, followed by cloning (where necessary), and sequencing (see Additional file 1).

Sequences were compared with publically available sequences using BLAST [14]. All malaria parasite sequences identified in this study exhibited 98-100% sequence identity to published sequences. To confirm species identification, a phylogenetic tree was built using a dataset comprising the sequences generated for this study and previously reported sequences from haemosporidian parasite species, including all *Plasmodium* species found in African great apes (see Additional files 1 and 2). Species names were assigned following the provisional taxonomy proposed in [3]. Sequences were deposited in EMBL under accession numbers: LK995432-LK995453. Content in mammal mitochondrial DNA from all samples was measured as described in [4].

### Statistical analysis

To test what influenced the probability of a malaria parasite infection, a generalized linear mixed model (GLMM) was run [15]. Into this model female status (pregnant *vs* non-pregnant), the number of days since conception or birth, and the age of the female were included as fixed effects. To account for the possibility that the impact of the number of days since conception or birth depends on whether the female is pregnant or not, the interaction between number of days since conception or birth and female status were also included as a fixed effect into the model. Finally, group size and season (see Additional file 1) were included as fixed effects and infant identification, mother identification and group as random effects to control for their potential effects. To control for the quantity of DNA in the sample, it was included as an offset term into the model. The model was implemented in R [16] using the function glmer of the R package lme4 [17]. P-values for the individual effects were determined using likelihood ratio tests (R-function drop1) [18]. The total sample size for this analysis was 384 data points (from 41 children of 27 mothers out of three groups) (see Additional file 1 for more details). The same model was also run using only the samples for which sequences were obtained (N = 21) as positive response for malaria parasite infection.

## Results

### Molecular analysis

Overall, 7.3% (N = 28) of the samples were positive for malaria parasites. This included 9% of the samples collected during pregnancy, and 4.9% of the samples collected during post-pregnancy. Of all females investigated, 48% were positive at least once. Sequences were obtained for 75% of the positive samples (N = 21). Malaria species sequenced included *Plasmodium gaboni* (N = 8), *Plasmodium reichenowi* (N = 5), *Plasmodium billcollinsi* (N = 5), *Plasmodium vivax* (N = 1), *Hepatocystis* sp. (N = 1), and one mixed infection (*P. gaboni* and *P. reichenowi*) (see phylogenetic tree in Additional file 2).

### Statistical model

**Figure 2:**
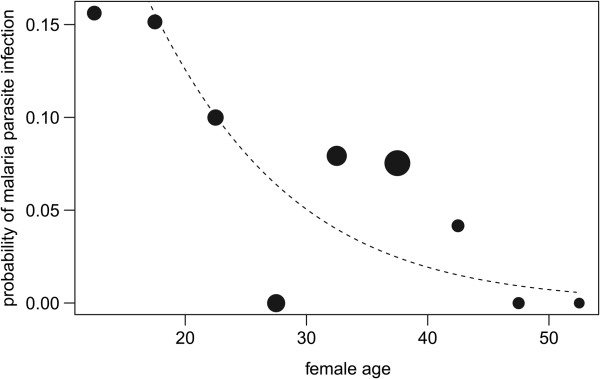
**Probability of malaria parasite infection as a function of female age.** The area of the points corresponds to the number of samples in the respective age class and the dashed line indicates the probability of malaria positivity per 200,000 units mitochondrial mammal DNA.

The full model was clearly significant as compared to the null model (likelihood ratio test: χ2 = 18.92, df = 4, P = 0.001). During pregnancy the probability of infection increased with the infant’s age (gestational age), but not obviously so after the infant’s birth (test of the interaction between days after birth or conception and mother pregnancy status: estimate + SE = 2.47 + 1.02, z = 2.42, P = 0.016; Figure 1). Moreover, the probability of infection decreased with mother’s age (estimate + SE = -1.00 + 0.43, z = -2.32, P = 0.02; Figure 2). Similar results were obtained with a more conservative set of positives (likelihood ratio test: χ2 = 13.44, df = 4, P = 0.009; interaction between days after birth or conception and mother pregnancy status: estimate + SE = 2.22 + 1.17, z = 1.91, P = 0.056; mother’s age: estimate + SE = -1.15 + 0.46, z = -2.48, P = 0.013). It is important to emphasize that by controlling for sample content in mammal DNA, it was ensured that none of these observations was driven by an increased quantity of (parasite-containing) blood or other body fluids in the feces.

## Discussion

Epidemiological studies in human populations have shown that prevalence of *Plasmodium* spp. infection (peripheral parasitaemia detected by *Plasmodium* spp. slide positivity [8, 19, 20] or by PCR [21]) and parasite density[8, 19, 20] are higher during pregnancy. Similarly, the results of the present study show that the probability of malaria parasite detection in adult female chimpanzees also increases during pregnancy. This suggests that, as in humans, pregnant female chimpanzees are more susceptible to infection or more likely to present higher parasitaemia (note that the latter explanation is favored to explain increased detection among pregnant women [9]). Of note, the peak of detection seems to occur at a different gestational age than in humans, e.g. towards the end of pregnancy in chimpanzees in contrast to the second [8] or first two [19] trimesters in humans.

There are two main explanations to the exacerbated susceptibility to malaria parasites seen in pregnant women: immune alterations and the existence of placenta-tropic malaria parasites [7]. Pregnancy is obviously associated with important hormonal changes which impact on the immune system. Secretion of cortisol, a well-known immunosuppressive hormone, continuously increases during pregnancy. Malaria infection and parasite load are positively related to cortisol levels in primigravid women [22], suggesting that an increased cortisol level effectively leads to decreased resistance to *Plasmodium*. This might be explained by this hormone's action on cell-mediated immunity, which, unlike humoral immunity, appears to be generally depressed during pregnancy. *De facto*, cell mediated immune response towards malaria antigens is down-regulated during pregnancy [23, 24]. The temporary impairment of the immune function (possibly mediated by cortisol and other pregnancy-associated hormones) might thus contribute to the increased susceptibility of pregnant women. Interestingly, pregnant chimpanzees also experience increased cortisol levels during gestation [25]. Cortisol reaches a peak concentration during late pregnancy [25], which is coincident with the increased probability of malaria parasite detection towards the end of pregnancy reported in this study. It is likely that cortisol increases result in immune impairment although this would obviously need further investigations. It is noted here that the non-invasive assessment of the immune function of wild chimpanzees, although being the only ethical option [26], will be extremely challenging.

In humans, the prevalence of malaria in pregnancy has been observed to decrease with increasing parity in women living in endemic areas (and therefore otherwise malaria-immune), which has led to the identification of a subset of *Plasmodium falciparum* parasites that express high levels of a variant surface antigen (*var*2CSA) allowing them to selectively target placenta [27]. Pregnant (particularly pauciparous) women might, therefore, face an enhanced susceptibility because they are naïve to this subpopulation of placenta-tropic parasites, against which they will only mount an effective immunity after several pregnancies. The relationship between peripheral blood parasitaemia and placental parasitaemia is not clearly established (they can occur simultaneously or not) [28] and exploring the occurrence of placental malaria in chimpanzees through placental blood analysis or histopathology will most likely reveal unfeasible. However, homologues of the *var*2CSA gene have been found in the genomes of malaria parasite species infecting chimpanzees, including *P. reichenowi* and *P. gaboni* [29, 30]. As *var* gene repertoires fluctuate considerably, including within malaria parasite species, e.g. only three of the ~60 *var* genes are found in all *P. falciparum* genomes [31], such a degree of conservation across great ape *Plasmodium* spp. may indicate selection-driven maintaining [29]. The observations reported here are compatible with (but no proof of) the notion that the adaptive advantage conferred by *var*2CSA to Laveranian parasites may have consisted in an increased access to pregnant females.

The decline in malaria parasite detection with increasing age of the females, also observed in humans [32, 33], confirms the influence of age on malaria susceptibility in chimpanzees [4]. The effect of maternal age might be combined with an effect of increasing gravidity on resistance to malaria parasites, similar to what is seen in humans [32, 33]. It is however difficult to distinguish one effect from the other due to the small number of positives in this study and the fact that, as an inherent consequence of the chimpanzees’ natural history, age and gravidity are strongly inter-related.

Knowing that pregnant female chimpanzees are less resistant against malaria parasite infection raises the question of the impact of the infection on pregnancy outcome, infant survival and, thus, general reproductive success of the chimpanzee community. So far, 49% of the infants included in this study died at an age below or equal to five years (nine of the currently living infants have not yet reach the age of five years) which is close to what was found before (44% according to Boesch and Boesch-Achermann [34]), and 24% died before reaching an age of one year. Three of the latter died of known causes other than malaria. Causes were unknown for six, and one was a stillbirth with malaria infection of the mother during the second trimester. If infections with AGA malaria parasites during chimpanzee pregnancy had consequences similar to malaria in pregnancy in humans - premature birth, low birth weight, enhanced risk of contracting malaria during infancy [10, 11] - it could be responsible for part of these mortalities. A model was run to test the effect of malaria infection of the mother during pregnancy on infant survival, which revealed a trend towards reduced survival, but results remained inconclusive due to the limited sample size and the instability of the model (see Additional file 1). Gathering more data on malaria parasite infection occurrence during chimpanzee pregnancy and on infant morbidity and mortality is clearly needed before reaching more definite conclusions. Thus far, a larger dataset could not be assembled, although the chimpanzee community living in the Taï National Park has been closely monitored for more than 30 years and non-invasive samples have been collected for more than 15 years.

## Conclusions

Together with a previous study on the effect of age on malaria infection [4], this study further indicates a similarity between the dynamics of malaria infection in chimpanzee and human populations from endemic areas, with young age and pregnancy constituting intrinsic risk factors for malaria infection. As anything affecting pregnancy ultimately affects reproductive success and infant survival, and therefore population health, whether malaria infection is, as in humans, associated with disease will be an important avenue of investigation.

## Electronic supplementary material

Additional file 1: **Supplementary information on molecular, statistical and phylogenetic analyses.** (PDF 97 KB)

Additional file 2: **Phylogenetic tree of a partial cytochrome** ***b*** **gene fragment from 81 haemosporidian parasites.** Bootstrap values are given above branches. This tree was mid-point rooted. Scale is in substitution per site. (PDF 317 KB)
