# Supplementary material for: Malaria parasite detection increases during pregnancy in wild chimpanzees
Source: Malar J. 2014 Oct 20;13:413. doi: 10.1186/1475-2875-13-413 (PMC4210475; doi:10.1186/1475-2875-13-413)
Supplement: Supplementary file 1 — Additional file 1: Supplementary information on molecular, statistical and phylogenetic analyses. (PDF 97 KB) [file 12936_2014_3572_MOESM1_ESM.pdf]

Additional file 1: supplementary information on molecular, statistical and phylogenetic analyses

Molecular analyses

Pan mammal PCR

Five µl of DNA extract was used to run a PCR targeting a 130 bp fragment of mitochondrial 16S gene (16Smam1 5 -CGGTTGGGGTGACCTCGGA-3' and 16Smam2 5 -GCTGTTATCCCTAGGGTAACT-3') in the presence of human and chimpanzee blocking primers (16Smam_blkhum3 5'-CGGTTGGGGCGACCTCGGAGCAGAACCC--spacerC3 and 16Smam_blksus1 5'-CGGTTGGGGTGACCTCGGAGTACAAAAAAC--spacerC3) (Calvignac Spencer *et al.* 2013 and references therein).

The PCR mixes contained 0.5 µl of each primer, 2.5 µl of each blocking primer, 2 µl dNTP (with dUTP replacing dTTP), 0.3 U AmpErase® uracil Nglycosylase (Invitrogen, Carlsbad, CA, USA), 2 mM MgCl2, 1X PCR buffer, 0.25 µl Platinum® Taq polymerase (Invitrogen) and assays were run under the following conditions: 7 min at 45°C, 10 min at 95°C, 42 cycles [30 s at 95°C, 30 s at 64°C, 60 s at 72°C], 10 min at 72°C. This was followed by sequencing of positive samples according to Sanger's methodology. Amplicons for which sequences were not clear were cloned using a Topo TA cloning kit.

Standard nested PCR for malaria parasites detection

The same protocol as for the nested qPCR described in De Nys *et al.* 2013 was used except for the second round which was run using Platinum® Taq (Invitrogen) according to manufacturer's instructions and under the following conditions: 5 min at 95°C, 40 cycles [30s at 95°C, 30 s at 57°C, 45 s at 72°C], 7 min at 72°C.

Statistical analysis

Generalized Linear Mixed Model

Season we included as the sine and cosine of the day (counted as number of days elapsed since Jan. 1st 1970) divided by 365.25 (to account for leap years). As random intercepts terms we included the ID of the mother, that of the infant (nested within mother ID) and also group. In order to keep type I error rate at the nominal level of 5% (Schielzeth & Forstmeier 2009; Barr *et al.* 2013) we included random slopes of pregnancy and mother age within mother as well as number of days since conception or birth within infant. We are aware that this is not the maximal model with regard to the random slopes possible (Barr *et al.* 2013). However, more random slopes terms were not possible to be included since otherwise the model did not converge anymore. Furthermore, among the theoretically possible random slopes those we included are biologically the most reasonable ones. We are aware that in principle one would need to control for potential autocorrelation in the response (more precisely in the residuals). However, due to the scarcity of positive samples (28 out of 385) and the facts that for some of the females we had only a few samples as well as for some of them being never determined positive this was not possible.

Prior to running the model we squareroot transformed number of days since conception or birth (to achieve a roughly symmetrical distribution) and then z-transformed number of days since conception or birth, mother age and group size to a mean of zero and a standard deviation of one. To control for the quantity of DNA in the sample we included it (log-transformed) as an offset term into the model. To test for the overall effect of the test predictors (females status, number of days since conception or birth, their interaction and female age) we compared the full model (Forstmeier & Schielzeth 2011) as described above

with a null model lacking these terms but comprising all other terms present in the full model using a likelihood ratio test (Dobson 2002). With regard to the assumptions, collinearity was no obvious issue (maximum Variance Inflation Factor determined from a standard linear model lacking the random effects: 1.31; (Quinn & Keough 2002; Field 2005)). We assessed model stability by excluding the levels of the different random effects one at a time, running the model for the derived reduced data sets, and comparing the estimated coefficients with those we got for all data. This revealed the model to be satisfactory stable (Table. S1).

Infant survival, Cox proportional hazards model

To test what influenced infant survival we ran a Cox proportional hazards mixed model (Therneau 2012). Into this we included mother malaria infection during pregnancy (positive vs. negative) and mother age at infant birth (to control for) as fixed effects. As random intercepts terms we included the ID of the mother and also group. We are aware that this is not the maximal model with regard to the random slopes possible (Barr *et al.* 2013); however, random slopes terms could not be included due to insufficient data (lack of variation of age and positivity within mother). To account for the sampling effort (number of samples per female and pregnancy) the model was weighted by this variable.

To test for the overall effect of the two fixed effects (mother positivity to malaria infection during pregnancy and mother age) we compared the full model as described above with a null model lacking these terms (Forstmeier & Schielzeth 2011) but comprising the same random effects as the full model using a likelihood ratio test (Dobson 2002). We implemented the model in R (version 3.0.2; R Core Team 2013) using the function coxme of the R package coxme (Therneau 2012). The total sample size for this analysis was 41 infants of 27 mothers out of 3 groups.

We assessed model stability by excluding the levels of the random effect of mother ID one at a time, running the model for the derived reduced data sets, and comparing the estimated coefficients with those we got for all data.

Overall, the full model was clearly significant as compared to the null model (likelihood ratio test: χ^2=34.16, df=2, P<0.001). We found a significant effect of mother age (estimate$\pm$SE=0.19$\pm$0.04, z=4.61, P<0.001), which was relatively stable (range of coefficients obtained from model stability checks: 0.082 to 0.964). This suggests that infant survival decreased with increasing age of the mother at infant birth. However this needs to be interpreted with caution as there was some degree of uncertainty, sample size was limited and the model might have been anti-conservative due to the lack of random slopes.

The effect malaria infection of the mother during pregnancy only showed a trend towards reduced infant survival when the mother was positive during pregnancy (estimate$\pm$SE=0.52$\pm$0.30, z=1.70, P<0.089), with a large degree of uncertainty (range of coefficients obtained from model stability checks: -0.38 to 4.59). The effect of positivity during pregnancy thus remained inconclusive, and a larger data set would be required to further investigate this question.

Table S1. Range of estimates obtained from running the Generalized Linear Mixed Model after excluding the levels of the different random effects one at a time.

**minima**	Estimate	SE	z	P	**maxima**	Estimate	SE	z	P
intercept	-6.516	0.652	-6.729	<0.001	intercept	-4.385	1.183	-5.102	<0.001
Pregnant	0.728	0.710	1.026	0.026	Pregnant	2.137	1.212	2.230	0.305
z.infant.age	-0.718	0.456	-0.927	0.354	z.infant.age	-0.132	1.025	-0.219	0.827
**z.mother.age**	**-1.374**	**0.398**	**-2.641**	**0.008**	**z.mother.age**	**-0.847**	**0.570**	**-1.857**	**0.063**
z.group.size	-0.298	0.375	-0.684	0.494	z.group.size	0.280	0.739	0.500	0.988
sin(season)	-0.551	0.455	-1.135	0.256	sin(season)	0.159	0.661	0.316	0.886
cos(season)	0.098	0.469	0.180	0.299	cos(season)	0.542	0.697	1.039	0.857
**Pregnant:z.infant.age**	**1.857**	**0.928**	**1.900**	**0.005**	**Pregnant:z.infant.age**	**3.86**	**1.727**	**2.779**	**0.057**

Phylogenetic analysis

Sequences from this study (N=22) were used together with previously published sequences of haemosporidian parasites (N=59) (Table S2) to build a phylogenetic tree (Figure S1 in Additional file 2) in order to confirm their identification. For this analysis, we used the 5' end of the cytochrome *b* gene that was covered by our confirmatory PCR. One hundred and thirty six sequences obtained from African great ape faecal samples by Liu *et al.* (2010) (mtDNA-3.4 kb, accession numbers HM235269–HM235404) were reduced to 25 haplotypes using Fabox v1.4.1 (Villesen 2007); the 34 sequences used in previous analyses by Blanquart and Gascuel (2011) were also included in our dataset. Sequences were aligned using Muscle (Edgar 2004) as implemented in Seaview v4 (Gouy *et al.* 2010). Models of nucleotide substitution were compared in a maximum likelihood framework; the Akaike information criterion (AIC) was used to select the model that offered the best fit/complexity trade-off (GTR+G). The maximum likelihood tree was then searched for using PhyML v3 (Guindon *et al.* 2010) using the selected model of nucleotide substitution and the BEST tree search algorithm. Branch robustness was estimated through non-parametric bootstrapping (500 pseudo-replicates).

Table S2. Sequences used for phylogenetic analyses.

**Strain**	**Accession No.**	**Reference**
P. falciparum	AY282930	Joy *et al.* 2003
P. gaboni	FJ895307	Ollomo *et al.* 2009
P. reichenowi	AJ251941	Conway *et al.* 2000
P. coatneyi	AB354575	Hayakawa *et al.* 2008
P. cynomolgi	AB434919	Hayakawa *et al.* 2008
P. fieldi	AB354574	Hayakawa *et al.* 2008
P. fragile	AY722799	Jongwutiwes *et al.* 2005
P. hylobati	AB354573	Hayakawa *et al.* 2008
P. inui	AB354572	Hayakawa *et al.* 2008
P. knowlesi	AY722797	Jongwutiwes *et al.* 2005
P. simiovale	AY800109	Mu *et al.* 2005
P. simium	AY722798	Jongwutiwes *et al.* 2005
P. vivax	NC_007243	Jongwutiwes *et al.* 2005
P. sP. DAJ-2004	AY800112	Mu *et al.* 2005
P. gonderi	AY800111	Mu *et al.* 2005
P. malariae	AB354570	Hayakawa *et al.* 2008
P. ovale	AB354571	Hayakawa *et al.* 2008
P. berghei	AF014115	Tan *et al.* 2000
P. chabaudi	AF014116	Tan *et al.* 2000
P. yoelii	M29000	Vaidiya *et al.* 1989
P. floridense	NC_009961	Perkins 2006
P. mexicanum	AB375765	Hayakawa *et al.* 2008
P. gallinaceum	AB250690	Omori *et al.* 2007
P. juxtanucleare	AB250415	Omori *et al.* 2007
P. relictum	AY733090	Beadell and Fleischer 2005
Ha. columbae	FJ168562	Perkins 2008
Pa. jb2.SEW5141	AY733087	Beadell and Fleischer 2005
Pa. jb1.JA27	AY733086	Beadell and Fleischer 2005
Pa. vireonis	FJ168561	Perkins 2008
L. caulleryi	AB302215	Omori *et al.* 2007
L. fringillinarum	FJ168564	Perkins 2008
L. majoris	FJ168563	Perkins 2008
L. sabrazesi	AB299369	Hirakawa *et al.* 2007
Hepatocystis sp.	FJ168565	Perkins 2008
*P.*_sp._G3-NDgor3203_SGA5.11	HM235382	Liu *et al.* 2010
*P.*_sp._G2-NKgor736_SGA5.6	HM235285	Liu *et al.* 2010
*P.*_sp._G2-NDgor3203_SGA5.4	HM235381	Liu *et al.* 2010
*P.*_sp._G2-NDgor3120_SGA5.5	HM235385	Liu *et al.* 2010
*P.*_sp._G2-LBgor185_SGA2.9	HM235282	Liu *et al.* 2010
*P.*_sp._G1-NKgor736_SGA5.9	HM235286	Liu *et al.* 2010
*P.*_sp._G1-GTgor34_SGA5.23	HM235308	Liu *et al.* 2010
*P.*_sp._G1-DSgor86_SGA5.1	HM235292	Liu *et al.* 2010
*P.*_sp._gorilla-DSgor86_SGA5.6	HM235311	Liu *et al.* 2010
*P.*_sp._C3-ONpts1321_SGA5.1	HM235387	Liu *et al.* 2010
*P.*_sp._C3-MBptt189_SGA5.10	HM235342	Liu *et al.* 2010
*P.*_sp._C3-GTptt722_SGA20.3	HM235325	Liu *et al.* 2010

**Strain**	**Accession No.**	**Reference**
P._sp._C3-BFpts1171_SGA5.7	HM235392	Liu *et al.* 2010
P._sp._C2-YBptt14_SGA5.12	HM235310	Liu *et al.* 2010
P._sp._C2-MTptt157_SGA5.1	HM235332	Liu *et al.* 2010
P._sp._C2-LUpts2078_SGA5.2	HM235320	Liu *et al.* 2010
P._sp._C2-LUpts2074_SGA2.13	HM235400	Liu *et al.* 2010
P._sp._C2-LBptt208_SGA5.11	HM235349	Liu *et al.* 2010
P._sp._C2-LBptt208_SGA5.7	HM235348	Liu *et al.* 2010
P._sp._C2-LBptt208_SGA5.4	HM235346	Liu *et al.* 2010
P._sp._C2-KApts1680_SGA30.11	HM235404	Liu *et al.* 2010
P._sp._C2-GTptt604_SGA5.12	HM235337	Liu *et al.* 2010
P._sp._C1-WEpte440_SGA5.11	HM235364	Liu *et al.* 2010
P._sp._C1-KApts382_SGA5.3	HM235402	Liu *et al.* 2010
P._sp._chimpanzee-LBptt208_SGA5.2	HM235345	Liu *et al.* 2010
*Hepatocystis*_F17.42_	LK995453	This study
P._sp._F17.70_	LK995441	This study
P._sp._F22.38_	LK995442	This study
P._sp._F32.19	LK995439	This study
P._sp._F35.2_	LK995446	This study
P._sp._F35.3__	LK995445	This study
P._sp._F74.19_	LK995434	This study
P._sp._F74.20__	LK995435	This study
P._sp._F74.25b	LK995437	This study
P._sp._F74.27__	LK995438	This study
P._sp._F74.34_	LK995436	This study
P._sp._F74.46_	LK995432	This study
P._sp._F74.49_	LK995433	This study
P._sp._F77.44__	LK995440	This study
P._sp._F293.10_	LK995451	This study
P._sp._F331.14-5_	LK995447	This study
P._sp._F331.14-8_	LK995448	This study
P._sp._F331.20_	LK995449	This study
P._sp._F331.21_	LK995450	This study
P._sp._F682.6_	LK995443	This study
P._sp._F682.8_	LK995444	This study
P._sp._F685.1_	LK995452	This study

References

Barr,D.J., Levy,R., Scheepers,C., Tily,H.J. 2013. Random effects structure for confirmatory hypothesis testing: Keep it maximal. J. Mem. Lang. 68

Blanquart,S., Gascuel,O. 2011. Mitochondrial genes support a common origin of rodent malaria parasites and Plasmodium falciparum's relatives infecting great apes. BMC Evol. Biol. 11, 70

Beadell, J.S., Fleischer, R.C. 2005. A restriction enzyme-based assay to distinguish between avian hemosporidians. J. Parasitol. 91(3), 683-5

Calvignac-Spencer,S., Merkel,K., Kutzner,N., Kuhl,H., Boesch,C., Kappeler,P.M., Metzger,S., Schubert,G., Leendertz,F.H. 2013. Carrion fly-derived DNA as a tool for comprehensive and cost-effective assessment of mammalian biodiversity. Mol. Ecol. 22, 915-924

Conway,D.J., Fanello,C., Lloyd,J.M., Al-Joubori,B.M., Baloch,A.H., Somanath,S.D., Roper,C., Oduola,A.M., Mulder,B., Povoa,M.M., Singh,B. and Thomas,A.W. 2000. Origin of Plasmodium falciparum malaria is traced by mitochondrial DNA. Mol. Biochem. Parasitol. 111 (1), 163-171

De Nys,H.M., Calvignac-Spencer,S., Thiesen,U., Boesch,C., Wittig,R.M., Mundry,R., Leendertz, F.H. 2013. Age-related effects on malaria parasite infection in wild chimpanzees. Biol. Lett. 9, 20121160

Dobson,A.J. 2002. An Introduction to Generalized Linear Models. (ed. C. Hall/CRC). Boca Raton

Edgar,R.C. 2004. MUSCLE: multiple sequence alignment with high accuracy and high throughput. Nucleic Acids Res. 32(5), 1792-1797

Field,A.P. 2005. Discovering statistics using SPSS : (and sex, drugs and rock 'n' roll), 2nd edn. Sage Publications, London ; Thousand Oaks, Calif

Forstmeier,W., Schielzeth, H. 2011. Cryptic multiple hypotheses testing in linear models: overestimated effect sizes and the winner's curse. Behav. Ecol. Sociobiol. 65, 47-55

Gouy,M., Guindon,S. & Gascuel,O. 2010. SeaView version 4 : a multiplatform graphical user interface for sequence alignment and phylogenetic tree building. Mol. Biol. and Evol. 27(2), 221-224.

Guindon,S., Dufayard,J.F., Lefort,V., Anisimova,M., Hordijk,W., Gascuel,O.2010. New Algorithms and Methods to Estimate Maximum-Likelihood Phylogenies: Assessing the Performance of PhyML 3.0. Syst. Biol. 59(3), 307-21

Hayakawa,T., Culleton,R., Otani,H., Horii,T. and Tanabe,K. 2008. Big bang in the evolution of extant malaria parasites. Mol. Biol. Evol. 25 (10), 2233-2239

Hirakawa,S., Omori,S., Sato,Y., Isobe,T., Yukawa,M. and Murata,K. 2007. Complete nucleotide sequences of the mitochondrial genome of Leucocytozoon sabrazesi. Unpublished. NCBI Direct Submission.

Jongwutiwes,S., Putaporntip,C., Iwasaki,T., Ferreira,M.U., Kanbara,H. and Hughes,A.L. 2005. Mitochondrial genome sequences support ancient population expansion in Plasmodium vivax. Mol. Biol. Evol. 22 (8), 1733-1739

Joy,D.A., Feng,X., Mu,J., Furuya,T., Chotivanich,K., Krettli,A.U., Ho,M., Wang,A., White,N.J., Suh,E., Beerli,P. and Su,X.Z. 2003. Early origin and recent expansion of Plasmodium falciparum. Science 300 (5617), 318-321

Liu,W., Li,Y., Learn,G.H., Rudicell ,R.S., Robertson,J.D., Keele,B.F., Ndjango,J.B., Sanz,C.M., Morgan,D.B., Locatelli,S., Gonder,M.K., Kranzusch,P.J., Walsh,P.D., Delaporte,E., Mpoudi-Ngole,E., Georgiev,A.V., Muller.M.N., Shaw,G.M., Peeters,M., Sharp,P.M., Rayner,J.C., Hahn,B.H.2010.Origin of the human malaria parasite *Plasmodium falciparum* in gorillas. Nature. 467, 420-425

Mu,J., Joy,D.A., Duan,J., Huang,Y., Carlton,J., Walker,J., Barnwell,J., Beerli,P., Charleston,M., Pybus,O.G. and Su,X.Z. 2005. Host switch leads to emergence of Plasmodium vivax malaria in humans. Mol. Biol. Evol. 22 (8), 1686-1693

Ollomo,B., Durand,P., Prugnolle,F., Douzery,E., Arnathau,C., Nkoghe,D., Leroy,E. and Renaud,F. 2009. A new malaria agent in African hominids. PLoS Pathog. 5 (5), E1000446

Omori,S., Sato,Y., Isobe,T., Yukawa,M. and Murata,K. 2007. Complete nucleotide sequences of the mitochondrial genomes of two avian malaria protozoa, Plasmodium gallinaceum and Plasmodium juxtanucleare. Parasitol. Res. 100 (3), 661-664

Perkins,S.L. 2006. Whole genome amplification and sequencing of mitochondrial DNA of saurian malaria parasites. Unpublished. NCBI Direct Submission.

Perkins,S.L. 2008. Molecular systematics of the three mitochondrial protein-coding genes of malaria parasites: corroborative and new evidence for the origins of human malaria. Mitochondrial DNA. 19(6), 471-8

Quinn,G.P., Keough, M.J. 2002. Experimental Designs and Data Analysis for Biologists. (ed. C.U. Press). Cambridge

R Core Team,R.C. 2013. R: A language and environment for statistical computing. (ed. R Foundation for Statistical Computing). Vienna, Austria

Schielzeth,H., Forstmeier, W. 2009. Conclusions beyond support: overconfident estimates in mixed models. Behav. Ecol. 20, 416-420

Tan,T.M.C., Noviyanti,R., Syafruddin, Marzuki,S. and Ting,R.C.Y. 2000. NCBI Direct Submission

Therneau,T. 2012. coxme: Mixed Effects Cox Models. R package version 2.2-3

Vaidya,A.B., Akella,R. and Suplick,K. 1989. Sequences similar to genes for two mitochondrial proteins and portions of ribosomal RNA in tandemly arrayed 6-kilobase-pair DNA of a malarial parasite. Mol. Biochem. Parasitol. 35 (2), 97-107

Villesen, P. 2007. FaBox: an online toolbox for fasta sequences. Molecular Ecology Notes, 7, 965–968
