# Supplementary figures and images for: Malaria parasite detection increases during pregnancy in wild chimpanzees
Source: Malar J. 2014 Oct 20;13:413. doi: 10.1186/1475-2875-13-413 (PMC4210475; doi:10.1186/1475-2875-13-413)

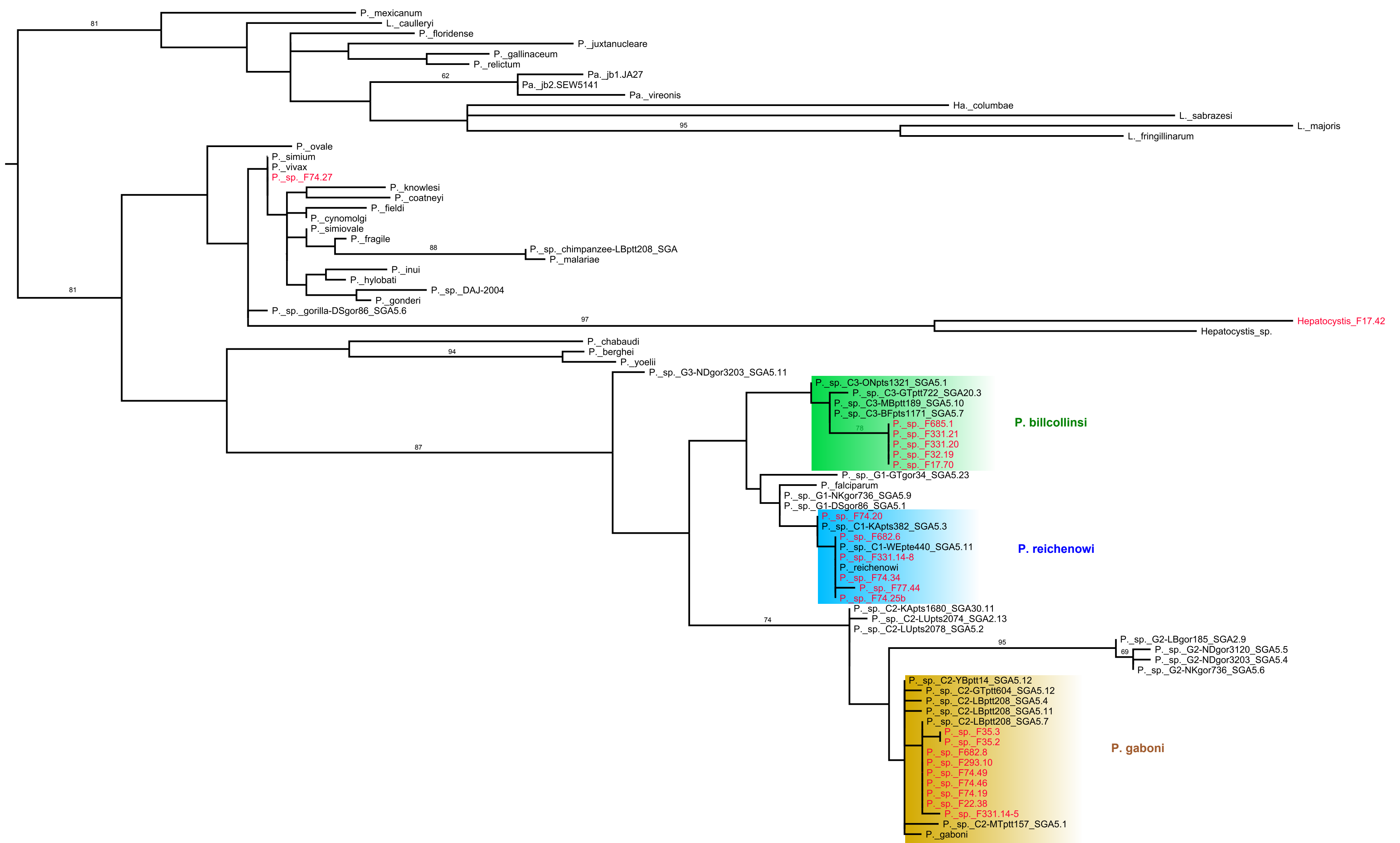

Supplement: Supplementary file 2 — Additional file 2: Phylogenetic tree of a partial cytochrome b gene fragment from 81 haemosporidian parasites. Bootstrap values are given above branches. This tree was mid-point rooted. Scale is in substitution per site. (PDF 317 KB) [file 12936_2014_3572_MOESM2_ESM.pdf]
